# Functional profiles of children with cerebral palsy in Jordan based on the association between gross motor function and manual ability

**DOI:** 10.1186/s12887-018-1257-x

**Published:** 2018-08-21

**Authors:** Nihad A. Almasri, Maysoun Saleh, Sana Abu-Dahab, Somaya H. Malkawi, Eva Nordmark

**Affiliations:** 1 Department of Physiotherapy, School of Rehabilitation Sciences, The University of Jordan, Queen Rania Al Abdallah St, Amman, 11942 Jordan; 2 Department of occupational therapy, School of Rehabilitation Sciences, The University of Jordan, Queen Rania Al Abdallah St, Amman, 11942 Jordan; 3 Faculty of Medicine, Lund university, P.0. 157, SE-221 00 Lund, Sweden

**Keywords:** Gross Motor Function Classification System-Expanded & Revised, Manual ability classification system, Cerebral palsy, Children, Functional profiles, Jordan

## Abstract

**Background:**

Cerebral palsy (CP) is the most common cause of physical disability in childhood. A major challenge for delivering effective services for children with CP is the heterogeneity of the medical condition. Categorizing children into homogeneous groups based on functional profiles is expected to improve service planning. The aims of this study were to (1) to describe functional profiles of children with CP based on the Gross Motor Function Classification System-Expanded & Revised (GMFCS-E & R) and the Manual Ability Classification System (MACS); and (2) to examine associations and agreements between the GMFCS-E & R and the MACS for all participants then for subgroups based on subtypes of CP and chronological age of children.

**Methods:**

A convenience sample of 124 children with CP (mean age 4.5, SD 2.9 years, 56% male) participated in the study. Children were classified into the GMFCS-E & R and the MACS levels by research assistants based on parents input. Research assistants determined the subtypes of CP.

**Results:**

Thirty six percent of the participants were able to ambulate independently (GMFCS-E & R levels I-II) and 64% were able to handle objects independently (MACS levels I-II). The most common functional profile of children with CP in our study is the “*manual abilities better than gross motor function*”. An overall strong correlation was found between the GMFCS-E & R and the MACS (*r*_*s*_ = .73, *p* < .001), the correlations vary significantly based on subtypes of CP and chronological age of children. A very strong correlation was found in children with spastic quadriplegia (*r*_*s*_ = .81, *p <* .001), moderate with spastic diplegia (*r*_*s*_ = .64, *p <* .001), and weak with spastic hemiplegia (*r*_*s*_ = .37, *p <* .001).

**Conclusions:**

The GMFCS- E & R and the MACS provide complementary but distinctive information related to mobility and manual abilities of children with CP. Subtypes of CP and chronological age differentiated functional profiles. Functional abilities of children with CP in Jordan have similar patterns to children with CP in other countries. Functional profiles can inform clinicians, researchers, and policy makers.

## Background

Cerebral palsy (CP) is the most common cause of physical disability in childhood [1]. A major challenge for delivering effective services for children with CP is the heterogeneity of the medical condition. Variety of clinical presentations can be observed in children with CP ranging from children who can ambulate and handle objects independently to children who have severe limitations in mobility and manual abilities further complicated by associated health conditions such as epilepsy and cognitive problems [2]. Therefore, it is useful to categorize children with CP into more homogeneous groups based on their functional profiles. The use of functional profiles in clinical sittings is expected to provide comprehensive description of abilities of children with CP which consequently may improve service planning and research.

Functional profiles of children with CP can be described utilizing functional classifications such as the Gross Motor Function Classification System- Expanded and Revised (GMFCS-E & R) [3, 4] and the Manual Ability Classification System (MACS) [5]. Functional classifications are consistent with the premises of the International Classification of Functioning, Disability, and Health (ICF) [6]. The ICF shifts the health professionals’ attention from focusing on primary motor impairments to functional activities and social participation which are considered the optimal outcomes of medical services for children with CP [6]. Functional classifications are useful in setting functional goals and planning services for children with CP in health care systems [3–5].

Associations between the GMFCS-E & R and the MACS allow description of functional profiles of children with CP [7]. The GMFCS-E & R is the first activity-based classification system that was developed to classify children with CP in five levels based on their current performance in gross motor function [3]. Later, the MACS was developed in order to classify manual abilities of children with CP [5]. Both classifications demonstrated acceptable reliability and validity in classifying children with CP and the GMFCS-E & R is reliable to be used in Arabic language [8]. The GMFCS-E & R and the MACS describe different but complementary motor functions and between them, GMFCS-E & R and MACS provide a good description of the functional profiles of children with CP [9]. The associations between the two functional classifications vary based on the subtypes of CP. Previous research found that the strength of associations among the GMFCS-E & R and the MACS based on subtypes of CP were strong to moderate for quadriplegia and hemiplegia and poor to fair for diplegia [10, 7, 11, 12].

Although the GMFCS-E & R and the MACS are available in Arabic language they are not being used in any clinical sittings in Jordan. Health professionals are accustomed to use the traditional impairment-based classification of CP than the functional classifications [13]. Consequently, rehabilitation services for children with CP are focused on treating impairments rather than improving activity and participation of children [13]. For example, physiotherapists who provide services for children with spastic quadriplegia focus on stretching and strengthening more than mobility and activity of daily living training [13]. Utilizing functional profiles might therefore provide a framework to classify children with CP based on their levels of function, and tailor rehabilitation services in Jordan towards outcome that are meaningful to children with CP and their families.

To our knowledge this is the first study in a low-income and middle-income country to describe functional profiles of children with CP based on GMFCS-E & R and MACS. Functional profiles are expected to shift the focus of rehabilitation in Jordan from impairment-based towards function-based services. In addition, functional profiles can be used to guide service planning and to allocate limited resources in areas of major needs of children with CP. We hypothesized that the gross motor function and manual abilities will vary within subtypes of CP and age groups. Variations between the GMFCS-E & R and the MACS by subtypes indicate the need to use functional classifications in addition to motor subtypes to accurately classify children with CP. Variations between the GMFCS-E & R and the MACS based on chronological age of children indicate the presence of a variety of functional abilities supporting the need to use more than one classification system to classify children with CP accurately. The aims of this study; therefore, were to (1) to describe functional profiles of children with CP based on the GMFCS-E & R and the MACS; and (2) to examine associations and agreements between the GMFCS-E & R and the MACS for all participants then for subgroups based on subtypes of CP and chronological age of children.

## Methods

### Participants

A convenience sample of 124 children 2 to 16 years of age (mean = 4.5, SD = 2.9 years, 56% male) participated in the study. All the participants had a medical diagnosis of CP confirmed by a neuropediatrician. Participant children were recruited from the major public hospital and the major public school in the capital city of Amman where the majority of children with CP receive rehabilitation services. Mothers’ of participant children mean age was 35 years (SD = 6.1) and fathers’ mean age was 37 years (SD = 7). Forty percent of the mothers and 46% of the fathers reported less than high school educational level. Table 1 presents demographic characteristics of participant children and parents.

**Table 1:** Participants’ characteristics

Variable (n)	Subcategories	n (%)
Age groups (*n* = 124)	2–4 years	59(47.6%)
	5–6 years	28(22.6%)
	> 6 years	37(29.8%)
Gender (n = 124)	Male	69 (55.6%)
	Female	55(44.4%)
Comorbidities (*n* = 123)	Vision impairment	45(36.6%)
	Hearing impairment	4(3.3%)
	Epilepsy/seizures	32(26%)
	Speech impairment	72(58.5%)
	Cognitive impairment	29(23.6%)
Mothers’ age (*n* = 121)		32.5 (SD = 6.1)
Mothers’ educational level (n = 123)	Less than high school	49(39.8%)
	Completed high school	38(30.9%)
	College (diploma 2 years)	19(15.4%)
	Graduate degree	15(12.2%)
	Postgraduate degree	2(1.6%)
Fathers’ age (*n* = 122)		37.9 (SD = 7.0)
Fathers’ educational level (n = 123)	Less than high school	56(45.5%)
	Completed high school	33(26.8%)
	College (diploma 2 years)	16(13.0%)
	Graduate degree	16(13.0%)
	Postgraduate degree	2(1.6%)

This study was approved by the Institutional Review Boards of the University of Jordan Hospital and the Ministry of Health. Participant families were recruited by their therapists and if they agreed to be contacted, a research assistant called the family and explained the study protocol. Each participating family were required to provide a written consent by one of the parents prior to data collection.

### Measures

#### Gross motor function classification system-expanded and revised (GMFCS-E & R)

The GMFCS [3] was developed to measure functional activities of children with CP. The GMFCS classifies children based on their gross motor function into five levels from Level I (walks without limitation) to Level V (severely limited mobility), as shown in Table 2. The system classifies current performance in daily life with focus on mobility rather than capabilities in standardized environments. The GMFCS was expanded and revised (GMFCS-E & R) [4] to include children with age 0–18 years and to reflect the potential impact of environmental and personal factors on children’s mobility. Content validity, inter-rater reliability, and test-retest reliability were established for children with CP [3, 14].

**Table 2:** Summary of GMFCS-E & R and MACS criteria

Level	GMFCS-E & R (Palisano et al., 1997)	MACS (Eliasson et al., 2006)
I	Walks without Limitations	Handles objects easily and successfully
II	Walks with Limitations	Handles most objects but with somewhat reduced quality and/or speed of achievement.
III	Walks Using a Hand-Held Mobility Device	Handles objects with difficulty; needs help to prepare and/or modify activities
VI	Self-Mobility with Limitations; May Use Powered Mobility	Handles a limited selection of easily managed objects in adapted situations
V	Transported in a Manual Wheelchair	Does not handle objects and has severely limited ability to perform even simple actions.

#### Manual ability classification system (MACS)

The MACS [5] describes children’s self-initiated manual ability to handle objects and their need for assistance or adaptation during daily manual activities. The MACS focuses on performance in home, school, community rather than capability in standardized environment. The MACS classifies children from Level I (handles objects easily and successfully) to Level V (doesn’t handle objects and has very limited ability to perform simple actions) as shown in Table 2. Construct validity and inter-rater reliability were established [5].

### Procedure

Upon obtaining the written consent, data were collected during the children’s visit to receive their physiotherapy treatments in hospital or during physiotherapy sessions in school. The GMFCS-E & R and MACS levels were determined by research assistants who were physiotherapists or occupational therapists with 3 to 5 years of clinical experience with parental input. Research assistants were criterion-tested to classify children reliably prior to data collection. The subtypes of CP were determined by the research assistants according to the topographical distribution and predominant type of motor disorder including: spastic hemiplegia (spasticity in one half of the body), spastic quadriplegia (spasticity in four limbs), spastic diplegia (spasticity in both lower limbs more than both upper limbs), dyskinesia (athetosis, dystonia, chorea), ataxia (hypotonia with dysmetria or poor balance), and unknown type.

### Data analysis

Statistical analyses were conducted using the Statistical Package for the Social Sciences (SPSS) for Windows, version 24.0 (SPSS Inc., Chicago, IL, USA). Analyses were performed for the entire sample first followed by subgroups based on the following topographical distribution of motor disorder: spastic diplegia, spastic quadriplegia, spastic hemiplegia, dyskinesia, ataxia, and unknown; and based on the children’s chronological age groups: two to less than four years, four to less than six years, and older than six years.

To achieve the first aim of the study descriptive analyses including frequency and cross tabulation of numbers of children in each level of the GMFCS-E & R and the MACS were performed to describe functional profiles.

To achieve the second aim of the study the following statistical tests were performed: (1) associations between GMFCS-E & R and MACS were examined by calculating Spearman’s Rho correlation coefficients (*r*_s_) because variables are ordinal. Spearman’s Rho coefficient (*r*_s_) was interpreted using the following criteria: *r*_s_ ≥ .8 very strong relationship; .6 ≤ *r*_s_ < .8 strong relationship; .4 ≤ *r*_s_ < .6 moderate relationship; .2 ≤ *r*_s_ < .4 weak relationship; *r*_s_ < .2 very weak relationship [15]; (2) Levels of agreement between GMFCS-E & R and MACS levels were assessed by calculating the non-weighted Kappa statistics. Kappa statistics were interpreted according to Altman criteria where kappa value of <.20 is poor, .21–.40 is fair, .41–.60 is moderate, .61–.80 is good and > .80 is a very good agreement [16]; and (3) Associations were examined based on subtypes of CP and children’s chronological age using Wilcoxon signed ranks test and Sign test. A probability level of *p* < .01 was considered statistically significant.

## Results

### Entire sample

Overall, a strong correlation was found between GMFCS-E & R and MACS levels (*r*_*s*_ = .73, *p* < .001) while the agreement between the two classifications was poor (kappa value = .19; SE = .05). Only 9% of the participants were able to ambulate independently and handle objects easily (Level I in both GMFCS-E & R and MACS), whereas 13% have severely limited mobility even with assistive devices and were unable to handle objects (Level V in both GMFCS-E & R and MACS). Of all participants, 36% were able to ambulate independently (GMFCS-E & R Levels I-II) and 64% were able to handle objects independently (MACS Levels I-II). Table 3 shows the distribution of participants across GMFCS-E & R and MACS levels. Fifty seven percent of the participants demonstrated manual abilities better than gross motor function and 34% have been classified into equivalent levels in both classifications (Wilcoxon signed ranks test: *p* < .001, Sign test: *p* < .001).

**Table 3:** Distribution of the participants across the GMFCS-E & R and the MACS levels

		MACS					Total
		Level I	Level II	Level III	Level IV	Level V	
GMFCS-E & R	Level I	11	5	0	0	0	16 (13.1%)
	Level II	16	9	3	0	0	28 (23.0%)
	Level III	4	10	1	0	0	15 (12.3%)
	Level IV	6	17	10	4	4	41 (33.6%)
	Level V	0	0	3	3	16	22 (18.0%)
Total		37 (30.3%)	41 (33.6%)	17 (13.9%)	7 (5.7%)	20 (16.4%)	122

### Groups based on subtypes of CP

**Fig. 1:**
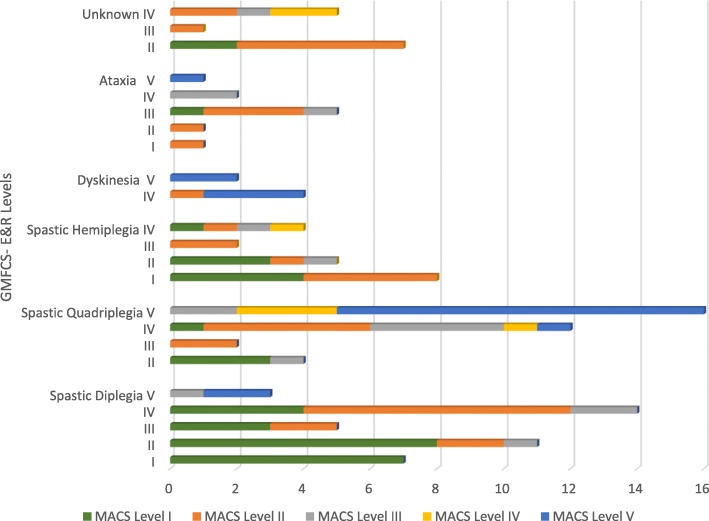
Distribution of the participant children between the GMFCS-E & R and MACS levels by subtypes of CP

Figure 1 shows distribution of children across GMFCS-E & R and MACS levels by CP subtypes. The relationship between GMFCS-E & R and MACS levels is differentiated by subtypes of CP. Specifically, correlations were very strong in children with spastic quadriplegia (*r*_*s*_ = .81, *p* < .001), strong in children with ataxia (*r*_*s*_ = .71, *p* < .001) and spastic diplegia (*r*_*s*_ = .64, *p* < .001), and weak in children with spastic hemiplegia (*r*_*s*_ = .38, *p* = .12) and dyskinesia (*r*_s_ = .32, *p* = .54). None of the participants had a mixed subtype of CP. Overall, poor agreement was found between GMFCS-E & R and MACS levels across different subtypes with kappa values <.2.

Table 4 illustrates concordance between GMFCS-E & R and MACS levels by different CP subtypes and chronological age of children. Only, children with spastic diplegia and spastic quadriplegia demonstrated different profiles of motor function. Seventy percent of children with spastic diplegia and 59% of children with spastic quadriplegia have better manual abilities than gross motor function (Wilcoxon signed ranks test: *p* < .001; Sign test: *p* < .001). However, in children with spastic hemiplegia, dyskinesia, and ataxia the relationships between the GMFCS-E & R and MACS levels were not differentiated significantly based on CP subtypes (Wilcoxon signed ranks test: *p* = .24, .71, .06; Sign test: *p* = .58, .63, .13).

**Table 4:** Concordance between the MACS and the GMFCS-E & R levels by subtypes of CP and chronological age groups

	MACS level < GMFCS- E & R level (Manual ability better than gross motor function)	MACS level > GMFCS-E & R level (Gross motor function better than manual ability)	MACS level = GMFCS-E & R level (Manual ability is similar to gross motor function)
Entire sample (*n* = 122)	69^**^ (56.6%)	12^**^ (9.8%)	41^**^ (33.6%)
Subtype of CP			
Spastic diplegia (*n* = 40)	28^**^ (70.0%)	1^**^ (2.5%)	11^**^ (27.5%)
Spastic quadriplegia (*n* = 34)	20^**^ (58.8%)	2^**^ (5.9%)	12^**^ (35.3%)
Spastic hemiplegia (*n* = 19)	8 (42.1%)	5 (26.3%)	6 (31.6%)
Dyskinesia (*n* = 6)	1 (16.7%)	3 (50.0%)	2 (33.3%)
Ataxia (*n* = 10)	6 (60.0%)	1 (10.0%)	3 (30.0%)
Unknown (*n* = 13)	6 (46.2%)	0 (0.0%)	7 (53.8%)
Chronological age			
2 - > 4 years (*n* = 58)	22^*^(37.9%)	10^*^ (17.2%)	26^*^ (44.8%)
4 - < 6 years (*n* = 28)	17^**^ (60.7%)	1^**^ (3.6%)	10^**^ (35.7%)
≥ 6 years (*n* = 36)	30^**^ (83.3%)	1^**^ (2.8%)	5^**^ (13.9%)

**Wilcoxon signed ranks teat and Sign test significant *p* < .001

*Wilcoxon signed ranks teat and Sign test significant *p* < .01

### Groups based on chronological age of children

**Fig. 2:**
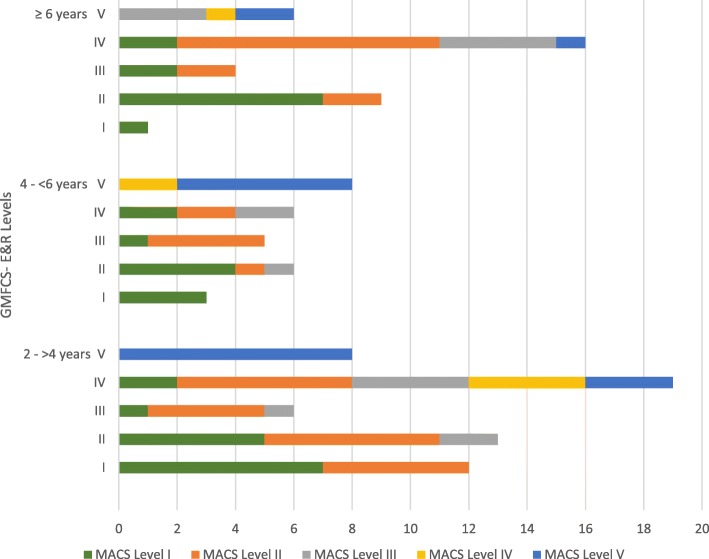
Distribution of the participant children between the GMFCS-E & R and the MACS levels by chronological age groups

Figure 2 shows distribution of participants across GMFCS-E & R and MACS levels by age groups. The strongest correlation was found between GMFCS-E & R and MACS levels of children at age four to less than six years (*r*_*s*_ = .81, *p* < .001). The agreement however was poor (kappa value = .21, SE = .10, *p* < 0.001) with 61% of the children demonstrated manual abilities better than gross motor function (Wilcoxon signed ranks test: *p* < .001, Sign test*: p* < .001). Strong correlation was found between GMFCS-E & R and MACS for children older than 6 years (*r*_*s*_ = .78, *p* < .001), with 83% demonstrating better manual abilities (Wilcoxon signed ranks test: *p* < .001, Sign test: *p* < .001). A strong correlation was also found (*r*_*s*_ = .73, *p* < .001) for the youngest age group with fair agreement (kappa value = .31, SE = .08, *p* < .001). Around 45% of the children were classified into equivalent GMFCS-E & R and MACS levels (Wilcoxon signed ranks test: *p* = .005, Sign test: *p* = .052).

## Discussion

This is, to our knowledge, the first study that describes functional profiles of children with CP and examines associations between the gross motor functions and the manual abilities in Jordan. The most commonly observed profile was “*manual abilities better than gross motor function*” which was demonstrated by 57% of the participants. Although associations between the GMFCS-E & R and the MACS were very strong in children with spastic quadriplegia and strong in children with spastic diplegia, and very strong in children less than four years and strong in children older than four years; agreements between the two classifications were poor across CP subtypes and chronological age groups indicating related yet diverse functional abilities of children with CP. Different functional profiles were observed and described based on subtypes of CP and chronological age of children.

Examination of functional profiles of children with CP in our study revealed that 53% of the participant children have severe limitation in gross motor functions (I.e., Levels IV and V GMFCS-E & R). This suggested that the majority of children who receive services in the public health care sector in Jordan have severe limitations in the gross motor functions. Describing functional profiles for children with CP in Jordan is expected to inform policy makers about the needs of children and their families. Implications for decision makers are to assure that public health care sector is being equipped to meet the extensive needs of children with severe limitation of function such as: wheelchairs, assistive devices, orthosis, and environmental modifications to enhance their functional activity and participation.

In congruence to research performed in other countries, we found a strong correlation yet poor agreement between the GMFCS-E & R and the MACS [7, 9–12]. This indicates that the two classifications complement each other and describe different types of activities of daily living which are ambulation and manual abilities of children with CP. The utilization of the two classifications by health professionals in Jordan is recommended to provide an accurate description of the functional performance of children with CP. Current rehabilitation practices in Jordan are based on traditional classifications of CP rather than functional classification, with focus on impairment rather than function-based interventions. Using functional classifications in practice is expected to shift the focus of rehabilitation professionals from impairment-based to functional-based practices, consequently, improving outcomes of services.

Functional profiles were differentiated based on subtypes of CP but the observed patterns were not consistent with the subtype’s definitions. The most common functional profile of children with diplegia was “*manual abilities better than gross motor function*”. This functional profile is consistent with the definition of diplegia, yet 30% of the children demonstrated other functional profiles. Also, the most common functional profile in the group of children with quadriplegia was “*manual abilities better than gross motor function*”. Although the term quadriplegia indicates involvement of both upper and lower extremities due to extensive injuries of the sensorimotor areas of the brain [7], 35% of children with spastic quadriplegia had equal fine and gross motor abilities, and 6% had gross motor functions better than manual abilities. These findings suggests that subtypes of CP include children with different functional abilities, highlighting the importance of using more reliable and accurate functional classifications to describe children with CP. A recommendation for health professionals is to combine traditional classifications with more reliable functional classifications when evaluating children with CP to provide comprehensive description of children with CP and guide service planning.

Functional profiles of children were differentiated based on children’s chronological age. Forty five percent of children with CP between two and four years of age demonstrated a profile of “*equivalent gross motor and manual abilities*”, whereas 61% of the children between four and six years of age and 83% of children older than six years demonstrated a profile of “*manual abilities better than gross motor function*”. This indicates that children in older age groups demonstrate better manual abilities than gross motor functions. Manual abilities and hand functioning require higher cognitive abilities and motor control than gross motor functions and occur at older ages which might explain increasing percentages of children in advanced manual abilities profiles in older age groups [9]. These findings should be interpreted with caution due to the convenience sampling method used in recruitment; most of the participants were recruited from physiotherapy clinics were children are usually referred due to gross motor function rather than manual abilities limitation. The influence of children’s age should be further examined with a population-based sample to confirm our findings.

Applications of functional profiles of children with CP can inform clinicians, researchers, and policy makers in Jordan. Clinicians can use the functional profiles to select appropriate treatment approaches based on child’s level of function, and to inform parents and help them to set up goals and plan for their children. For example children with better functional profiles (i.e. better gross motor and fine motor abilities) are more likely to function well in everyday activities in home, school, and community requiring services that are more focused on participation outcomes and integration in the community. Whereas children with limited functional profiles (i.e. profiles of limited gross motor and fine motor abilities) are more likely to demonstrate limitations in activity and require more assistance requiring more intensive treatment plans that focuses on activities of daily living and independency [17, 18]. The use of functional classifications among clinicians can improve communication and coordination of services. Researchers can use functional profiles in clustering children with CP in homogeneous groups to conduct focused intervention studies. Policy makers can use functional profiles to anticipate needs of children with CP and their families and to insure availability, accessibility, and coordination of services required to fulfill these needs.

This study shows important strengths in that participant children were all classified by criterion tested physio- and occupational therapists with parents’ consensus on classifications levels. In addition, participant children had the GMFCS-E & R and the MACS levels determined by the same therapist. The results of the study should be considered in light of some limitations in relation to sample size and selection of participants which might limit generalization of results. This also raises the need for population-based sample to be able to examine the national profiles of children with CP in Jordan and allow for international comparisons.

## Conclusions

Health professionals in Jordan are encourage to use both the GMFCS-E & R and MACS in addition to traditional subtypes classification in order to classify children with CP with focus on function rather than impairment. Both the GMFCS-E & R and the MACS provide complementary but distinctive information related to mobility and handling of children with CP, supporting the need to use the two classification to provide comprehensive description of abilities of children with CP. Functional profiles of children with CP provide a practical and easy way for assessment to plan for services, guide provision of interdisciplinary and comprehensive services for children with CP, and enhance communication among professionals who provide services for children with CP and their families in Jordan.

## Abbreviations

CP: cerebral Palsy
GMFCS-E & R: Gross Motor Function Classification System Expanded and Revised
ICF: International Classification of Functioning, Disability, and Health
MACS: Manual Abilities Classification System
